# Laser Treatment for Diabetic Retinopathy: History, Mechanism, and Novel Technologies

**DOI:** 10.3390/jcm13185439

**Published:** 2024-09-13

**Authors:** Siyu Wang, Rui Hua, Yuqi Zhao, Limin Liu

**Affiliations:** Department of Ophthalmology, The First Affiliated Hospital of China Medical University, No. 155 Nanjing Bei Street, Heping District, Shenyang 110001, China or 20091136@cmu.edu.cn (R.H.);

**Keywords:** diabetic retinopathy, retinal laser therapy, laser photocoagulation, anti-VEGF, real-world conditions, diabetic complication

## Abstract

**Background**: Diabetic retinopathy (DR), as a complication of diabetes mellitus (DM), remains a significant contributor to preventable vision impairment in the working-age population. Laser photocoagulation is essential in treating DR in conjunction with anti-vascular endothelial growth factor (VEGF) injection, steroids, and vitrectomy. This review summarizes the history of laser photocoagulation and highlights its current role and long-term effectiveness in real-world conditions. **Methods**: The National Clinical Trial (NCT), PubMed, Google Scholar, and China National Knowledge Infrastructure (CNKI) databases were searched utilizing combined or individual keywords, and a total of 121 articles were reviewed by the authors. **Results**: Several novel laser photocoagulation technologies, such as patterned scanning laser, subthreshold micropulse laser, navigated laser, multimodal imaging-guided laser, and retina rejuvenation therapy, substantially decrease the adverse effects and improve the accuracy and security of laser therapy. Numerous studies have demonstrated the outstanding clinical efficacy of combination therapies with pharmacologic treatments like anti-VEGF in treating DR and diabetic macular edema (DME). A 20-year follow-up retrospective study in our center preliminarily demonstrated the long-term effectiveness of conventional laser photocoagulation. **Conclusions**: More clinical trials are required to confirm the clinical effectiveness of novel laser technologies. Better treatment protocols for the combination therapy may be detailed. Anti-VEGF treatment has better effects, especially for DME and in a short period. But in real-world conditions, given the long-term effectiveness and economic advantages of conventional laser treatment, it should be prioritized over anti-VEGF injection in certain situations.

## 1. Introduction

Diabetic retinopathy (DR) is a microvascular complication of diabetes mellitus (DM), which remains an essential contributor to preventable vision impairment in the working-age population. DR is usually subtle and asymptomatic at the early stages, and if not treated promptly, it will lead to irreversible vision impairment [1,2]. The pathophysiology of DR involves many hypotheses, including generating mediators and angiogenic factors. These will lead to alternation in blood flow, capillary permeability, and acellular capillary formation, which results in retinal edema and ischemia of the retina, clinically defined as nonproliferative DR (NPDR). As ischemia increases enough to lead to neovascularization, it develops into proliferative DR (PDR). It can further progress into hemorrhage and retinal detachment (RD) as the neovascular grows into the vitreous. Diabetic macular edema (DME) is a maculopathy that occurs when leakage happens in the macula [3]. The classification of DR and DME by the Ophthalmological Society of Chinese Medical Association is shown in Table 1 [4].

The treatment of DR can be categorized into systemic therapy, laser photocoagulation therapy, pharmacotherapy, and surgical therapy. Retinal laser photocoagulation therapy can be completed in a clinic or operating room during surgery. For several decades, it has been well established to reduce the high-risk development of DR [5,6]. Many modified laser therapies have sprung up in recent years, such as pattern laser and subthreshold laser photocoagulation. In addition to laser photocoagulation, other treatments such as controlling blood glucose levels, anti-vascular endothelial growth factor (VEGF) therapy, steroids, and vitrectomy have also been widely used and provide satisfactory clinical efficacy. Other therapies, like peroxisome proliferator-activated receptor alpha (PPAR-α) agonists, traditional Chinese medicine, as well as nanotechnology, also should not be ignored in DR treatment [7,8,9].

Retinal laser photocoagulation therapy in treating DR has developed over the years to minimize complications and patient discomfort while maintaining or improving the therapeutic effects. This review aims to summarize the history of retinal laser photocoagulation therapy, summarize the emerging technologies, and emphasize the current role and considerations in real-world conditions when intravitreal injection has become prevalent. The use of laser treatment for other conditions, like posterior capsule opacification, tumors, and retinal detachment, is not included.

## 2. Materials and Methods

This review concludes with articles on the laser treatment of DR and DME. The PubMed database, National Clinical Trial (NCT) database Google Scholar, and China National Knowledge Infrastructure (CNKI) database were utilized in searching the relevant literature from 2004 to 2024 via the following keywords: diabetic retinopathy, retinal laser, laser photocoagulation, laser therapy, diabetic macular edema, anti-VEGF, and combined treatment. The keywords were utilized alone and in combination, and additional articles were identified by reviewing the reference lists. To ensure completeness and avoid redundancy, we included a total of 121 relevant articles following the criteria below, and all authors comprehensively examined the articles included in the reference list.

Inclusion criteria: (1) published from January 2004 to January 2024; (2) language is limited to Chinese and English, or at least with English abstract; (3) manually screened relevant to our topic.

Exclusion criteria: (1) similar to more complete or updated studies; (2) unable to be fully accessed; (3) pre-print articles; (4) deemed unsuitable for the review.

## 3. History of Retinal Photocoagulation Therapy

Meyer-Schwickerath introduced the concept of retinal photocoagulation in the 1940s. The sunlight photocoagulator was developed in 1947 and successfully treated a patient with malignant eye tumors. In 1949, he changed the light source to Beck-arc (a high-intensity arc) and the xenon arc photocoagulator was developed to treat eye tumors and vascular diseases [10]. In 1960, stimulated optical radiation in ruby was first found, and it was able to make an exceptionally bright, monochromatic, highly directional beam [11]. Ruby laser photocoagulation has been demonstrated to be successfully used in treating many ocular diseases. Still, it has limited efficacy in some retinal vascular diseases, like neovascularization, due to the red output beam [12].

In 1969, based on the discovery of the argon, krypton, and xenon ion lasers, L’Esperance introduced the high-power argon laser photocoagulator. The argon laser, compared to krypton or xenon, could produce powerful radiation with 488 nanometer (nm) and 514 nm wavelengths in the blue-green spectral region, which were the deciding factors in regular selecting the argon laser as the basis for the source for the photocoagulator [13]. From then on, the argon blue-green laser became mainstream in ophthalmology for its effectiveness in retinal vascular disease despite a relatively weaker penetration than red light (620–750 nm) [14]. With the widespread clinical application of laser, the argon green laser stood out from the crowd for minimal absorption by xanthophyll (mainly in the macula) but a relatively high absorption for melanin [15]. The yellow laser shares a similar therapeutic effect to the green laser but with several additional advantages such as less energy requirements, a wider therapeutic window, and less discomfort [16,17]. However, the expense and size limited its clinical application until a more compact and cheaper semiconductor diode laser (yellow, 577 nm) was developed [18].

The neodymium-doped yttrium aluminum garnet laser (Nd:YAG) was first tested in 1971 by L’Esperance. The Nd:YAG laser utilizes an yttrium aluminum garnet crystal with a dopant of triply ionized neodymium, which emits a laser at 1064 nm. The visible green laser at 532 nm was obtained by frequency doubling, and this highly collimated, intense and monochromatic beam has several advantages over the argon laser (541.5 nm), such as the solid state in design, smaller size, and ease of maintenance [19].

The semiconductor diode laser was introduced in 1962 and was characterized by being compact, with relatively high cost-effectiveness, and with exceptionally long operating lifetimes [20]. However, due to the relatively low power outputs, its clinical application had been limited until the development of high-power gallium-aluminum-arsenide (GaAlAs) semiconductor devices emitting infrared wavelength near 800 nm [21]. Because of the relatively low absorption of 800 nm infrared laser by retinal and choroidal tissue, increased exposure and intensity were required to achieve similar photocoagulation effects, which would cause more patient discomfort [22]. To overcome these shortcomings, various visible wavelength diode lasers are now available in clinical practice, such as IQ 532 and IQ 577 (IRIDEX Corporation, Mountain View, CA, USA), which emit green and true-yellow lasers, respectively [23].

In addition to the above mainstream retinal laser, the tuneable dye laser is similar to argon and krypton lasers in retinal photocoagulation and iridectomy. Other commonly used lasers like carbon dioxide (CO2), Holmium: YAG (Ho:YAG), neodymium-doped yttrium lithium fluoride (Nd:YLF), and erbium: YAG (Er:YAG) lasers will not be discussed in this review, and more details are available in other articles [24].

## 4. Mechanism of Conventional Retinal Laser Photocoagulation

Thermal retinal damage and protein denaturation caused by light absorption by target pigment tissue on the retina is the basic principle that achieves therapeutic effects. This damage results in permanent chorioretinal scars. The scar’s width, depth, and recovery time depend on the grades of the laser energy.

The widely accepted primary mechanism of retinal laser photocoagulation therapy is that the destruction of photoreceptor cells by photocoagulation decreases the oxygen demand. The replacement of the glial scar allows more oxygen from the choriocapillaris to diffuse to the inner retina to relieve hypoxia. With the rise of oxygen tension, vasoactive factors such as VEGF and protein kinase C (PKC) decrease, and neovascularization is inhibited. Adequate oxygen tension relieves the constriction of the retinal arteries, decreases hydrostatic pressure in capillaries, and relieves retinal edema [25,26]. Meanwhile, oxygen from the choroid can penetrate the inner retina through the laser scar, which is a mechanism only when the laser lesion is intense enough to reach the inner nuclear layer [27].

Another theory hypothesizes that retinal laser photocoagulation may decrease the neovascularization stimulator levels in eyes by directly destroying relevant cells. Meanwhile, scar tissue formed during the retina repairment may release potential neovascularization inhibitors, such as transforming growth factor beta (TGF-β), erythropoietin (EPO), and pigment epithelium-derived factor (PEDF) [28,29].

Conventional focal laser in treating retinal edema is hypothesized to impede the leaking microaneurysms which lead to cytokine release and macula edema reabsorption during retina recovery [30]. Hypoxia relief, oxygenation improvement, and retinal tissue autoregulation reduction resulting in retinal artery dilation had been assumed as a mechanism of grid laser photocoagulation contributing to relieving macular edema [31]. Other mechanisms of novel laser therapy are discussed in the corresponding section.

## 5. Conventional Laser Photocoagulation

Scatter or panretinal photocoagulation (PRP) is the most often used conventional laser photocoagulation for treating DR. The long-term therapeutic effect of protecting retinal and choroidal neurovascular situations has been proved [32]. The transpupillary laser is the most common route, which can be delivered to the retina through a slit lamp using a contact lens in the clinic room or endo-laser photocoagulation in the operating room during the surgery. A laser indirect ophthalmoscope helps to treat patients with physical or mental defects who cannot sit at the slit lamp [33]. These transpupillary laser delivery systems need an articulating arm containing mirrors and an optic cable connected to the laser light source device.

**Figure 1:**
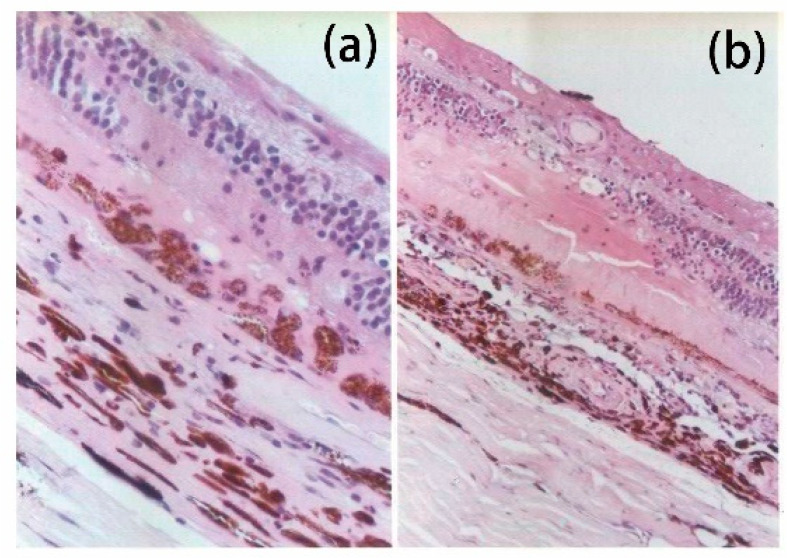
(**a**) The histological sections of grade II laser lesions; (**b**) the histological sections of grade III laser lesions [37].

Typical laser powers for PRP range from 100 to 750 milliwatts (mW). The spots’ diameter ranges from 200 to 500 micrometers (μm) and durations vary between 100 and 300 milliseconds (ms) to reach medium-intensity burns (Tso grade III laser lesion). Each spot should be separated by at least half to one diameter, leaving at least 500 μm distant from optic nerve head and 3000 μm from macula. In total, 1000 to 2000 visible gray-white spots are applied in the peripheral retina. The complete treatment regimen should be segmented into 2 to 4 cycles with 2 to 4 week intervals to reduce the chances of side effects and discomfort, or in one session via endo-laser during surgery [31,34]. Studies have reported that due to the high intensity of treatment received in a single-session completed with PRP during operation, it may lead to an increased risk of complications such as macular edema [35]. Laser lesions can be divided into five grades according to Mark O. M. Tso. The Tso grade III laser lesion was further divided into mild, moderate, and severe, as shown in Table 2 [36]. The histological sections of grade II and III laser lesions are shown in Figure 1.

Due to the destructive mechanism of PRP, it led to several side effects and disadvantages. Patient discomfort, divided treatment sessions, impairment of peripheral vision field, color and night vision, and difficulty adjusting to dim or bright lighting and distance judgment are the most frequent complaints mentioned by patients [38]. Acute elevated intraocular pressure (IOP) was reported within hours after the laser photocoagulation [39]. Retinal fibrosis, choroidal detachments, cystoid macular edema, macular pucker, and vitreous bleeding of neovascularization and retinal detachment were also reported in some articles [31,40]. Among these complications, macular edema is noteworthy as it could affect the visual outcome of the patients. Previous studies have shown that the incidence of macular edema after PRP was about 25–43%, with persistent chronic macular edema occurring up to 8% (average follow-up of 14 months) [41,42]. The mechanism was unclear, but it was generally believed to be caused by the burn-induced destruction of the blood–retinal barrier and the increase in cytokine release [35]. Multiple-session treatment, extending the treatment interval, reducing the intensity of laser spots, novel laser technologies [42,43], and combining pharmacologic therapies such as steroid and anti-VEGF agents [41,44,45] have been demonstrated to be effective in preventing and dealing with this condition and improving visual prognosis.

The Diabetic Retinopathy Study (DRS) (1758 patients, 1972 to 1975) was a large randomized controlled trial (RCT). It found that PRP could significantly decrease the likelihood of severe vision impairment by at least 50% in treating high-risk PDR. High-risk was defined as PDR with (1) active neovascularization, (2) vitreous or preretinal hemorrhage, or (3) neovascularization greater than or equal to one-fourth to one-third of the disc area within one disc diameter (DD) of the optic nerve [46]. The study’s last report emphasized three factors that need to be considered before PRP: the likelihood of vision impairment, the benefit from PRP, and the risk of other adverse effects [5]. The Early Treatment Diabetic Retinopathy Study (ETDRS) (3711 patients, 1979 to 1989, 22 centers) was a representative and multicenter clinical RCT aiming to find out the clinical efficacy of focal/grid laser photocoagulation in treating DME and the timing to initiate laser photocoagulation surgery [47]. The study demonstrated that focal/grid laser photocoagulation with visible burn decreased the risk of vision impairment by at least 50% in DME or retina edema, making it a benchmark treatment protocol [48]. Direct focal laser photocoagulation was applied to treat focal edema. The moderate intensity spots were from 50 to 100 μm in size and were exposed for between 50 and 100 ms. The whitening of the microaneurysm or other leakage is the focal laser photocoagulation treatment’s endpoint. Grid laser photocoagulation was applied to treat diffuse edema. The 50 to 200 μm sized spot’s intensity should be above the threshold but less intense than usual PRP spots, and it leave at least one spot width apart with an exposure duration between 50 and 100 ms [49,50].

The mild macular grid (MMG), as well as the Modified Early Treatment Diabetic Retinopathy Study (mETDRS) protocol, are studies aiming to verify a new focal/grid laser photocoagulation approach which could decrease the incidence of some side effects like scar extension resulting in significant vision loss and central scotomas [30,51]. The mETDRS technique utilized a modified laser photocoagulation, which is less intense than conventional focal/grid laser photocoagulation. The treating areas remained unchanged, but the endpoint of treatment was modified so that no change in color was required [52]. The MMG technique utilized a lighter intensity and more widely diffused grid laser photocoagulation throughout the macula, but microaneurysms were not photocoagulated. Clinical RCT demonstrated that retinal thickness was reduced more by the modified ETDRS laser photocoagulation by measured optical coherence tomography (OCT). These two modified techniques had fewer unexpected adverse effects than the classic ETDRS techniques [30].

## 6. Pattern Scanning Laser Photocoagulation

Based on the conventional retinal laser treatment, by improving the exposure duration, wavelength, spot space and size, innovations emphasize maintaining or enhancing the therapeutic effect while minimizing adverse side effects resulting from retinal tissue damage. Table 3 shows the parameters, merits, and demerits for several laser techniques.

The PASCAL photocoagulator (PAtterned SCAnning Laser, 514 nm wavelength argon ion laser, Lumenis Corporation, Santa Clara, CA, USA) was a semiautomated patterned scanning laser photocoagulation system with more precision and safety than conventional laser photocoagulation. It demonstrated a novel method of laser photocoagulation utilizing a variety of laser spot patterns (4 to 56 spots) created by a scanner to deliver rapid and predetermined sequenced laser pulses rather than individually placing a series of single spots [53]. With the help of the semiautomated scanning pattern and the shorter pulse durations (10–30 ms), PASCAL significantly improved efficiency, increased safety and accuracy and eased patient discomfort [54]. Shorter pulse duration burns have been proven to have less tissue damage, especially to the inner retinal layer. Histologic studies found that the nerve fiber layer and retinal neuron layer remained intact after photocoagulation using shorter pulse duration, which resulted in less patient pain, better recovery, and fewer side effects [55]. Multi-point scanning matrix laser photocoagulation is another patterned scanning laser system that can be achieved by a photocoagulator like MC-500VIXI (multicolor, 532 nm, 577 nm, 647 nm, diode laser, NIDEK Corporation, Aichi, Japan) [56].

The spot of pattern scanning laser photocoagulation should 200 to 400 μm in size, with 10 to 20 ms exposure duration, and it should achieve grade III burns. The spot grid pattern can vary from 3 × 3 to 7 × 7 arrays. A clinical RCT compared PRP to pattern scanning laser photocoagulation in treating PDR or severe NPDR, and results demonstrated that pattern scan laser photocoagulation had similar efficacy of lesion regression with less collateral tissue damage than the conventional PRP [57]. However, this trial only had six months of follow-up, and the long-term efficacy is unascertainable. In a retrospective comparative study, pattern scanning laser was proven to be less effective than traditional PRP in treating PDR (73% persistence of neovascularization via PASCAL and 34% via traditional argon PRP, P = 0.0008). The number of relevant articles is limited, and it is recommended to change treatment parameters; for example, increasing the number, spot size, or exposure duration when using PASCAL for high-risk PDR [58].

Pattern scan laser photocoagulation has also proven highly effective for reducing retinal and macular edema and minimizing scar formation. The ring or arc pattern templates can safely and accurately be applied on the central foveal, excluding the avascular zone. The barely visible laser spots with a 100 μm size and 10 ms exposure duration should be reduced by 25 mW after reaching the gray-white modified ETDRS threshold (PASCAL, 532 nm, Nd:YAG, Optimedica Corporation, Santa Clara, CA, USA) [59]. A retrospective observational trial in 2016 reported that the therapeutic effectiveness and safety of PASCAL laser (532 nm, 10 to 20 ms exposure, Optimedica Corporation, Santa Clara, CA, USA) is similar to conventional argon laser photocoagulation in treating DME [60].

## 7. Subthreshold Micropulse Laser Therapy

Subthreshold micropulse laser (SMPL) is a relatively less damaging therapy which limits the damage in the retinal pigment epithelium (RPE). It is also known as selective retinal therapy (SRT) [61]. The conventional laser photocoagulation having a “threshold” of visible grayish spots indicates the treatment endpoint, which means the laser thermal damage is high enough to reach the retinal neuroepithelium layer to change the transparency of the inner retina layer. In contrast, SRT is a subthreshold laser that avoids any visible intra-retinal damage [62]. To achieve this kind of “subthreshold” therapeutic effect, the pulsed laser beam was utilized, and it was proven that the shorter the duty cycle (DC) is, which is equivalent to a longer interval between consecutive pulses, the smaller the pulsed laser lesions [31].

The biological and physiological mechanisms that lead to therapeutic effects could be explained in many ways. A study demonstrated that RPE cells can generate some factors inhabiting neovascularization after photocoagulation, similar to the TGF-β [63]. Other theories assumed that the photocoagulation improved the RPE barrier and pump function to resolve the edema or the induced RPE up-and-down regulation of factors that regulate neovascularization and vascular leakage [64]. There are two classic application modes for SRT. The large area–pulsed mode protocol uses a series of short micropulse laser with parameters of 0.2 to 1.0 μs exposure duration, 100 to 500 pulses, 100 to 500 Hertz (Hz) rate, 160 μm size, and 70–100 μJ power [65]. Another protocol is the small spot–continuous wave scanning mode, which utilizes 40 μm and 66 μm sized spots with 6.6 m per second (m/s) in speed or 18 μm with 10.6 m/s in speed (PASCAL, 532 nm Nd:YAG laser, Optimedica Corporation, Santa Clara, CA, USA) [66].

The pulsed mode protocol SRT was validated to be secure and efficacious in treating DME with multiple optional retreatments and less adverse effects in conventional laser photocoagulation, like vision loss, scotomas, and discomfort due to the undamaged nerve fiber layer, cone and rod cells, and choroid [67,68]. The optimal treatment time for SRT photocoagulation is when the thickness of the central macular is less than 400 μm [69]. Other reports demonstrated that the therapeutic effects of SRT treating DME via lasers with different wavelengths showed no significant differences, and no treatment consensuses are there to guide clinical practice [70].

The subthreshold diode micropulse (SDM) is another SRT protocol; it utilizes a series of submillisecond pulses to extend interval periods between each laser micropulse and avoid causing permanent tissue destruction. The 810 nm light can be absorbed more and diffuses less to the inner neurosensory retina than other wavelengths [71]. Typically, a series of laser micropulsea 75 μm in size and exposed for 200 to 400 ms is applied 125 to 200 μm outside the fovea, with a DC of 100 μs exposure duration and a 50 to 150 μs interval, and the power is 150 to 1200 mW in total [61]. The SDM has been shown to have equal or superior clinical effect compared to the modified ETDRS or MMG protocols in treating DME [72,73]. A non-comparative trial demonstrated that the SDM laser has a clinically significant effect for at least three years in treating DME [74].

Despite its validated safety and efficacy, SRT is not widely adopted for ophthalmic clinical utilization due to the invisibility of the subthreshold laser spots [66]. Therefore, many techniques like optoacoustic (OA), optical-feedback techniques (OFT), and temperature-controlled photocoagulation (TCP) were developed to assist the SRT threshold determination [75]. Endpoint management (EPM) is designed to help choose therapeutic laser energy. Briefly, it is a titration algorithm that obtains a power spectrum for each patient. A baseline laser energy level that is just sufficient to produce a barely visible threshold lesion should be first ascertained. Subsequently, the power spectrum will be obtained by changing the laser power, which ranges from the subvisible retinal laser burns limited primarily to RPE to the intense photocoagulation of the inner retina tissue [76]. Many clinical trials have proven that SRT utilizing this algorithm achieved satisfactory effectiveness and enhanced safety (PASCAL 532 nm green and 577 nm yellow wavelength laser source) [77,78].

Based on the EPM algorithm, a novel treatment regimen called the “sandwich technique” has been proven to be an effective modified therapy that utilizes a traditional subthreshold short-pulse duration laser and an EPM-assisted laser in treating DR and DME. In this treatment protocol, subthreshold laser spots were applied around the parafoveal area, positioned 500 μm from the macular center, and with approximately 1000 EPM laser spots applied across the macula about 6 mm in diameter, while leaving a margin of 300 μm around the macular center [79]. However, this approach still has its shortcomings; for example, the power spectrum could vary greatly based on the different retina performing titration algorithms, and an experienced ophthalmologist is needed to ensure its effectiveness.

## 8. Image-Guided Navigated Laser Delivery System

Several essential clinical novel laser treatments will become mainstream in retina practices over the next few years, aiming to relieve the patient’s discomfort, reduce the treatment difficulty, and decrease the risk of complications.

The navigated laser photocoagulator (NAVILAS; OD-OS Inc., Teltow, Germany) is a prototype of an image-guided eye-tracking laser therapy system. NAVILAS allows the incorporation of live fundus imaging, including but not limited to fundus fluorescein angiography (FFA) photographs, fundus autofluorescence (FAF) photographs, and color fundus images. It integrates laser photocoagulation protocols of the retina, like grid and pattern scan laser photocoagulation. NAVILAS is advantaged in terms of retina navigation, allowing for high-precision and high-speed laser spot application with theoretical errors of less than 60–110 μm via the computer assistance systems. It utilizes a slit-based scanner, which can take 25 high-contrast and -sharpness fundus images per second during the treatment and provide a 50° or 85° view field for focal or panretinal laser treatment [80,81]. Clinical trials have shown that NAVILAS is equally or at least not less safe and effective in treating DR and DME via panretinal and focal laser photocoagulation [82]. When combining NAVILAS and anti-VEGF therapy, the required injection times were significantly reduced compared to monotherapy [83]. Benefiting from the pre-operation planning of the laser spot’s placed area and pattern before laser photocoagulation and the computerized navigation system, NAVILAS is a highly time-efficient system in contrast to conventional PRP or pattern scanning laser [84]. While considering accuracy and safety, it can achieve neater and more uniform spots with less discomfort via reducing treatment duration [85]. Benefiting from the fundus image presented on a computer screen, there is no need for the slit lamp, but at the same time, a stereoscopic view is unavailable.

## 9. Targeted Retinal Photocoagulation 

Targeted retinal photocoagulation (TRP) is a laser protocol that only applies laser spots to the non-perfusion retina. Reddy, in 2009, first came up with this treatment plan in order to decrease the risk of side effects of conventional PRP in treating DR [86]. The ultra-widefield fluorescein angiography (UWFFA) and ultra-widefield Swept Source OCT angiography (UW SS-OCTA) are of great value for SRT in accurately assessing the treatable retinal leakage areas and non-perfused areas [87]. A pilot study demonstrated that UWFFA-assisted TRP (PASCAL, 20 ms, 1500 spots) had a respectable safety profile and clinical efficacy for the treatment of DR [88]. Compared with conventional PRP, TRP can relieve a similar level of retinal neovascularization and with less retinal function damage. Due to the reduction in the total number of laser spots, the treatment duration is shorter, and the associated discomfort is less than that of conventional PRP [89]. Like other novel laser treatments, more clinical RCTs are needed to demonstrate TRP’s long-term therapeutic efficacy. Whether the limited treatment at retinal leakage areas and non-perfused areas may lead to disease progression or require further laser supplementation treatment needs to be further considered in clinical practice.

## 10. Multimodal Imaging-Guided Laser Therapy

The multimodal imaging-guided laser therapy needs a multimodal image integrated system to perform a more precise and less invasive laser photocoagulation. In 2018, Takamura first reported the merged image-guided laser photocoagulation (MIG-PC) method. Photoshop CS6 (Adobe Inc., San Jose, CA, USA) was utilized to incorporate fundus images, including color fundus images, OCT, and FFA images [90]. This novel laser treatment system needed ophthalmologists to manually incorporate patients’ fundus images on the computer before the laser treatment began, and until recently, Head Up Display-1 (HUD-1) was developed based on the PASCAL system. HUD-1 frees operators from diverting their attention from the slit lamp microscope to the monitor to obtain the reference image of the treatment target area, therefore increasing efficiency and guaranteeing safety [91].

## 11. Retina Rejuvenation Therapy

Nanosecond laser or retina rejuvenation therapy (2RT) is an innovative laser system that selectively applies a micropulse laser on the RPE with a 3 ns pulse duration. Like SRT, the 2RT laser limited the damage in RPE and caused no damage to the neural retinal layer, photoreceptors, or Bruch’s membrane. It has been demonstrated that 2RT can induce the generation of matrix metalloproteinases (MMPs) and PEDF which leads to proliferation and retiling of treated RPE [92]. A pilot study showed that 2RT has a positive therapeutic effect in treating DME but has relatively low risk of adverse effects caused by focal/grid photocoagulation [93]. More clinical RCTs are needed to confirm 2RT’s effectiveness before widespread clinical applications.

## 12. Photo-Mediated Ultrasound Therapy 

Photo-mediated ultrasound therapy (PUT) is a novel, high-precision, non-invasive anti-vascular method utilizing synchronized ultrasound bursts and nanosecond laser irradiation. By changing the wavelength of the laser beam, PUT can selectively target different tissues. In animal models, PUT has been proven to have a unique advantage in treating eye diseases associated with microvessels and neovascularization without damaging surrounding neurosensory tissues, like retinal and choroidal neovascularization [94,95]. PUT has significant potential for clinical translation, and further clinical and safety trials are required.

## 13. Photobiomodulation Therapy

Photobiomodulation therapy (PBT) is a novel treatment characterized as being highly precise, non-invasive, safe, and cost effective; it is mainly aimed at the adjunct treatment of DME. PBT can utilize near-infrared light (660–1000 nm) to enhance mitochondrial function, counteract inflammation, enhance supporting cell function, and decrease microvascular leakage. Many clinical studies have demonstrated that PBT is a potential adjunct treatment for DME, but limited by the small sample size, further studies of this therapeutic option are warranted [96]. A multicenter, clinical RCT, Diabetic Retinopathy Clinical Research Network (DRCR) Protocol AE demonstrated that the efficacy of PBT in treating DME with good vision is not well confirmed, despite its high tolerance and safety profile [97]. The role of PBT in treating DR and DME still needs to be determined, and standard treatment patterns need to be further refined.

## 14. Compared and Combined with Pharmacologic Therapies

Recently, more and more articles recommend the combination of anti-VEGF agent intravitreal injections and laser treatment, especially for early PDR and severe NPDR combined with DME. Almost all these research outcomes favor anti-VEGF for better visual outcomes and the regression of neovascularization [98]. The combination therapy of anti-VEGF intravitreal injections and conventional laser photocoagulation has been proven to achieve satisfactory efficacy and safety in DR treatment by improving best-corrected visual acuity and neovascularization regression, with no potential increased incidence of adverse effects [99].

DRCR.net is a cooperative website coordinating many multicenter clinical trials to improve the care of people with DR and DME. Over the past two decades, it helped demonstrate that anti-VEGF agents are effective as an alternative treatment for PRP in treating DR and as first-line therapy for eyes with DME [100]. DRCR Protocol B, a multicenter clinical RCT involving 840 eyes, found out that focal/grid laser photocoagulation is more safe and effective than triamcinolone acetonide (TA) injection [101]. DRCR Protocol H was a clinical RCT, the objective of which was to provide clinical outcomes of bevacizumab intravitreal injection in treating DME; it was shown that bevacizumab intravitreal injection has similar therapeutic effect to focal laser photocoagulation in a short follow-up period. However, the safety and effectiveness conclusions were limited, and a phase III randomized clinical trial was needed [102]. Then came the DRCR Protocol S, a five-year, phase III clinical RCT, which evaluated the effect and safety of ranibizumab intravitreal injection combined with deferred laser photocoagulation and prompt laser photocoagulation as a monotherapy. The study found that PDR treated with ranibizumab had no statistically significant differences from PRP treatment, resulting in visual acuity at five years. But the risk of vision impairment and visual field impairment was lower in the group treated with ranibizumab. However, the difference between groups diminished as time went on [103]. The other details of the studies can be accessed on their website (https://public.jaeb.org/drcrnet/stdy, accessed on 9 June 2024).

At present, there are many anti-VEGF agents available in the clinic for selection, like ranibizumab, conbercept, aflibercept, brolucizumab, and faricimab [104,105]. Due to the improvement in both the functional and anatomical aspects of the retina, anti-VEGF intravitreal injection is now more accepted than conventional PRP in treating DR and DME. Several studies have shown that anti-VEGF drugs combined with PRP therapy have a certain benefit in vision improvement compared with monotherapy, and are superior to laser photocoagulation alone in vision improvement for CI-DME (center-involving diabetic macular edema); this makes them suitable for becoming the first-line DME treatment [106,107,108,109]. However, strict adherence to follow-up and timely and regular injections are needed to preserve the therapeutic effect [110]. It is reported that the cessation of anti-VEGF intravitreal injection can lead to the recurrence or exacerbation of neovascularization [111]. And about 20 to 50% of DME eyes had accepted laser therapy as a rescue treatment due to poor response to anti-VEGF or treatment interruption [112].

Similarly, some studies focus on the comparison of the efficacy of other pharmacologic therapies, like steroids compared to laser photocoagulation, or in combination. For instance, steroid agent intravitreal injection is suggested to be a second-line therapy for the treatment of refractory DME [113]. The dexamethasone (DEX) intravitreal implant (Ozurdex, Allergan Inc., Irvine, CA, USA) was demonstrated to have a long-lasting therapeutic effect for the treatment of DME, which can provide continual release of DEX into the vitreous cavity for up to 6 months [114]. Meanwhile, the eye complications brought about by steroids should not be overlooked, such as complicated cataract and high IOP [115]. It is worth noting that some experiments showed that the combination therapy of laser and steroids can lead to some undesired side effects. A rabbit model study showed that TA injection combined with laser photocoagulation would lead to a broader laser burning scar, especially if more intense laser photocoagulation is needed [116].

## 15. Laser Photocoagulation in Real-World Conditions

Since the emergence of anti-VEGF agents, their use instead of conventional laser photocoagulation as the primary treatment for DR and DME has been a subject of ongoing debate. Timely laser photocoagulation is effective for preserving sight and preventing severe vision loss, but it is hardly effective in reversing visual loss [117]. There is an abundance of research demonstrating the extraordinary efficacy and safety of anti-VEGF agents intravitreal injection. When it comes to a real-world condition, a careful choice is required when accepting this expensive and invasive therapy, which needs regular injection and may, albeit rarely, lead to endophthalmitis [118]. All these PDR or DME treatment plans that rely on regular anti-VEGF agent injections may not achieve the expected therapeutic efficacy and may even lead to a catastrophic consequence compared to an eye treated with PRP, like vitreous hemorrhage or neovascular glaucoma if follow-up is somehow ceased [119]. Many personal, social, psycho, and financial factors may also lead to significant lapses in the follow-up to injection, resulting in irreversible blindness, such as pandemic, cardiovascular accidents, loss of critical illnesses, economic hardship, or simply noncompliance [120]. 

Considering these practical limitations, laser treatment combined or alone can offer the advantage of minimizing the need for an intravitreal injection with anti-VEGF agents while providing a highly effective and durable therapeutic effect in treating DR. However, anti-VEGF injection may be superior when patients are anxious about their vision-related quality of life after laser photocoagulation. Thus, ophthalmologists should tell patients about available treatment options and collaborate with them to determine the most suitable therapeutic approach. It is also worth noting that a considerable number of DR patients who need PRP treatment develop nephropathy about nine years after PRP. Thus, ophthalmologists should not only focus on the eyes, but the consultation between different clinical specialties should also be emphasized. For DR patients who are young, have poor renal function, high blood sugar levels, and are in the early stages of DM, it is advisable to enhance follow-up [121].

## 16. Discussion

Over the years, several novel laser photocoagulation technologies have emerged based on conventional laser photocoagulation and have achieved equal or non-inferior clinical efficacy in treating DR and DME. All those modified technologies either reduce the side effects like the impairment of peripheral vision field, scotoma, and patient discomfort, or lower the operation difficulty to improve accuracy and security, or both simultaneously. Pattern scanning laser photocoagulation increases safety and accuracy while decreasing the time required and patient comfort during PRP treatment. SRT and micropulse laser photocoagulation avoided unnecessary damage to the neurosensory retinal tissue, maintaining efficacy but with fewer adverse effects, especially in treating DME. And with the help of the EPM algorithm, the safety of subthreshold (invisible) laser photocoagulation was guaranteed. The NAVILAS further improved the accuracy, security, and efficiency of PRP treatment. 2RT and PBT provide a new therapeutic idea that shows potential in the treatment of DME. These modalities related to retinal regeneration are expected to compensate for the limitations of laser therapy in reversing vision loss. PUT is a high-precision, anti-vascular treatment that has achieved uplifting outcomes in animal experiments, but further clinical and safety trials are expected. All these novel laser photocoagulation technologies represent significant progress in the treatment of DR and DME, but before their randomized controlled phase III clinical trial is completed, PRP will not be challenged as the first choice for laser treatment of DR. Meanwhile, due to the lower intensity of these novel laser technologies, none of them can achieve a laser scar tunnel that assists choroidal oxygen diffusion to the retina. The laser scar in the conventional PRP, which helps oxygen penetration, is a unique mechanism that neither the novel laser technologies nor anti-VEGF management can access, which may contribute to long-lasting treatment efficacy. Our clinical center is currently collecting and organizing clinical data from about 4000 patients with DR who have undergone PRP from 2005 to 2024. All laser treatments involved were accomplished by one ophthalmologist following the same treatment standards. This retrospective study aims to further confirm the long-term efficacy of laser treatment through a large sample size and extended follow-up duration, while analyzing factors influencing laser efficacy in the treatment of DR and DME. Moreover, the time span of this study encompasses numerous significant time points, such as the emergence of novel laser techniques, anti-VEGF agents, and the COVID-19 pandemic. The study is now in the stage of data collection and processing, and the preliminary results confirm the long-term therapeutic effect of PRP in the treatment of DR.

In terms of wavelength, the dominance of the green laser over the blue-green laser is now being challenged by the yellow laser, which is superior in safety, comfort, and broader clinical applications. The diode lasers are gradually replacing the argon laser and Nd:YAG laser due to their more compact size, relatively high efficiency, and long lifetimes.

For the last few years, despite the widespread use of anti-VEGF and steroid agents in treating DR and especially DME, laser treatment remains an important therapy due to the cost considerations and potential risks of loss to follow-up in real-world conditions. Conventional retinal laser photocoagulation like PRP can provide an economical, reliable, and long-lasting treatment efficacy. Although PRP may lead to some irreversible and unnecessary tissue damage, it can prevent the progression of DR into severe vision-threatening complications such as vitreous hemorrhage and neovascular glaucoma. Additionally, it reduces the numbers needed for anti-VEGF injections during the combined treatment, minimizing the risk of complications and exacerbating progression in cases of loss to follow-up. Focal and grid laser photocoagulation combined with PRP can provide a longstanding therapeutic effect for DEM without sequential anti-VEGF intravitreal injections despite lower visual acuity, and this sacrifice is worth it in certain conditions. The economic burden and risks associated with frequent intravitreal injections warrant consideration. In real-world conditions, PRP remains an important treatment for severe NPDR and PDR, because of its durable efficacy, relative safety, better economy, and less invasive nature, despite some drawbacks.

## 17. Conclusions

For many decades, laser photocoagulation has remained the mainstay of treatment for DR and is now considered as a second-line therapy of DME. When providing intravitreal anti-VEGF and corticosteroid agent treatment, doctors should value not only the perfect effectiveness but also the real-world conditions. Future studies may emphasize the better treatment protocols for the combination therapy. Although anti-VEGF drugs have achieved significant success, we cannot slow down the exploration of novel laser technologies, which aim to achieve better visual acuity with fewer adverse effects for years to come.

## Figures and Tables

**Table 1:** The classification of DR and DME by the Fundus Disease Group of Ophthalmological Society of Chinese Medical Association in 2022.

Disease	Stage	Fundus Pathology
DR	I	Only microaneurysms.
	II	Severity more than I but less than III.
	III	One of the following findings: (1) ≥20 intraretinal hemorrhages in each quadrant; (2) Venous beading in ≥2 quadrants; (3) IRMA in ≥1 quadrant.
	IV	NVE or NVD.
	V	Retinal fibrosis, with or without preretinal or vitreous hemorrhage.
	VI	TRD combined with FVM.
DME	NCI-DME	Retinal thickening not within 1 mm diameter from the fovea.
	CI-DME	Retinal thickening within 1 mm diameter from the fovea.

DR: diabetic retinopathy; DME: diabetic macular edema; IRMA: intraretinal microvascular abnormalities; NVE: neovascularization elsewhere; NVD: neovascularization at the disc; TRD: tractional retinal detachment; FVM: fibrovascular membrane; CI-DME: center-involving DME; NCI-DME: no center-involving DME.

**Table 2:** The grading of laser lesions according to Mark O. M. Tso.

Laser Grade	Histologically Change within 24 h	Clinical Appearance	Clinical Application
I	RPE swollen and vacuole formation	Faint, grayish-white discs	Leakage lesion like CSCR and CME
II	RPE, photoreceptor layer and ONL necrosis; choriocapillaris occluded by thrombi	A grayish ring around a denser whitish center	No therapeutic value
III	RPE, photoreceptor layer, ONL and INL necrosis	Two distinct grayish rings surrounding a white center	Ischemic, proliferative retinopathy like DR, RVO
Mild	Slight damage in INL	A soft white center	
Moderate	Moderate damage in INL	A dense white center	
Severe	Severe damage in INL	An even dense white center	
IV	Full-thickness retinal necrosis	White rings surrounding a dense white center	Chorioretinal tumor

RPE: retinal pigment epithelium; CME: cystoid macular edema; CSCR: central serous chorioretinopathy; INL: inner nuclear layer; ONL: outer nuclear layer; RVO: retinal vein occlusion.

**Table 3:** Comparison of serval laser parameters in the treatment of PDR and DME.

Laser Techniques	Duration (ms)	Spot Size (μm)	Indications	Advantages	Limitations
PRP	100 to 300	200 to 500	DR	Mature technology, long-term efficacy	More side effects
Focal/Grid	50 to 100	50 to 200	DME	Repeatable treatment	Limit indication, unexpected injury to the macula
Pattern Scanning	10 to 1000	100 to 400	PDR/DME	Short treatment time and less pain	Unascertained long-term efficacy, without eye tracking
SMPL/SRT			DME	Limit the damage in RPE	Invisible laser spots
Pulsed mode	2 × 10^−4^ to 1 × 10^−3^	160			
Scanning mode	CW (6.6 or 10.6 m/s)	18 or 66			
SDM	0.01 to 0.03	75			
Navigated	10 to 1000	100 to 400	PDR/DME	Short treatment time, eye tracking	Without stereoscopic view
TRP	100 to 300	200 to 500	DR	Less tissue damage	May require further laser supplementation
Multimodal Imaging-Guided	100 to 300	200 to 500	DR	More precise and safe	Manually incorporate images
2RT	3 × 10^−6^	400	DME	Less tissue damage	No treatment protocols

PRP: panretinal photocoagulation; DR: diabetic retinopathy; DME: diabetic macular edema; PDR: proliferative DR; CW: continuous wave; SMPL: subthreshold micropulse laser; SRT: selective retinal therapy; SDM: subthreshold diode micropulse; TRP: targeted retinal photocoagulation; 2RT: retina rejuvenation therapy.
